# The cardiovascular profile of soccer referees: an echocardiographic study

**DOI:** 10.1186/1476-7120-6-8

**Published:** 2008-02-12

**Authors:** G Galanti, A Pizzi, M Lucarelli, L Stefani, M Gianassi, V Di Tante, L Toncelli, A Moretti, F Del Furia

**Affiliations:** 1 Postgraduate School of Sports Medicine, Sports Medicine Laboratory Dept Emergency Medicine, University of Florence, Italy; 2 FIGC Federazione Italiana Gioco Calcio - AIA Associazione Italiana Arbitri, Italy

## Abstract

**Background:**

During a soccer game, the cardiovascular system is severely taxed The referees must be alert and their level of fitness must be such that fatigue will not impair their decision-making. Referee's peak overall performance is usually after 40 when the performance starts to decline. We evaluated the morphological and functional cardiac profile of professional soccer referees.

**Materials and methods:**

We submitted to a clinical and echocardiographic exam a group of 120 professional soccer referees aged 25 – 45 years, including the first division of the Italian Championship, matched with 120 soccer players, including élite soccer players. Data were compared using an unpaired Student's t test. Statistical significance was with p < 0.05.

**Results:**

Right ventricle dimensions (22.2 ± 3.8 vs 25.9 ± 2.4 mm) and Left Ventricular Mass Index (LVMi) (100.5 ± 45.2 vs 105.4 ± 17.3) were significantly greater in referees than in active soccer players. Left atrium dimensions (33.7 ± 8.9 vs 36.2 ± 3.1 mm), aortic root (29.7 ± 7.9 vs 32.1 ± 3 mm) and LVMi (115.1 ± 16.7 vs 134.1 ± 19.9 g/m^2^) were significantly greater in élite soccer players than in first-division referees.

**Conclusion:**

Our investigation shows that right ventricle is greater in referees than in soccer players. The differences (left atrium, aortic root and LVMi) between first division referees and élite soccer players may derive from the different training workloads.

## Introduction

During a soccer game the cardio-circulatory system is severely taxed [1], and soccer referees need to have high levels of physical fitness. To referee a soccer game, they must be alert and near the scene of action, and their level of fitness must be such that fatigue will not impair their decision-making. Referees are often subjected to proportionally increasing physical demands as years go by. Indeed, their peak performance is usually between 30 and 45 years of age, when cardiovascular athletic performance starts to decline. Several studies have investigated the athletic performance of referees [2-4] The aim of our study is to evaluate the morphological and functional cardiac profiles of professional soccer referees.

## Methods

### Subjects

We examined 120 professional Italian soccer referees from the Italian Association of Referees, 35 officiated in first-division, aged between 25 and 45 years, over the period 7^th ^to 15^th ^July 2006, before the start of the soccer season.

The referees were compared with 120 soccer players (35 élite soccer players from the first division) examined at the Sports Medicine Centre of the University of Florence. Furthermore, first-division referees were matched with first-division soccer players.

Referees generally train 3–4 times/week and soccer players 5–6 times.

The qualities which are mainly worked on are: aerobic power, strength, sprint resistance (the athlete runs at a velocity > 23 km/h and for a distance < 50 m), speed (velocity > 23 km/h and distance < 30 m). Table 1–2.

**Table 1:** First-division soccers' training sessions

**FD Soccers**	Number/YEAR Mean ± Standard Deviation	Number/WEEK Mean
Training sessions	296 ± 10	5.6
Aerobic power	73 ± 9	0.8
Strength	84 ± 12	2.1
Speed	77 ± 6	1.2
Sprint resistance	69 ± 4	1.3

**Table 2:** First division referees players' training sessions

**FD Referees**	Number/YEAR Mean ± Standard Deviation	Number/WEEK Mean
Training sessions	125 ± 14	3.6
Aerobic power	40 ± 6	1
Strength	35 ± 9	1
Speed	30 ± 7	0.9
Sprint resistance	27 ± 5	0.8

All procedures described in the present study were approved by our Local Ethics Committee.

### Clinical evaluation

All referees and players underwent a general clinical examination, which included history- taking, a general clinical check-up, and a cardiological examination consisting of echocardiography and ECG at rest.

### Echocardiography

**Figure 1:**
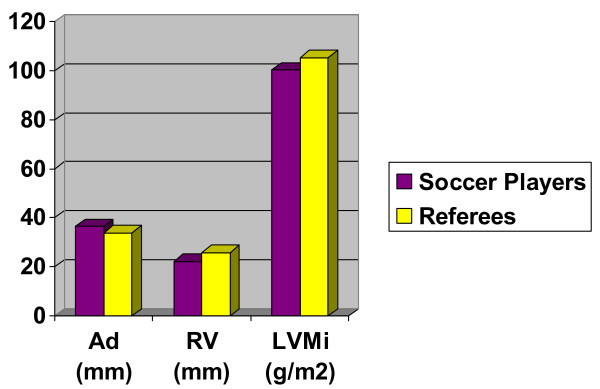
Histogram summarizing the echo-findings of the study "referees vs soccer players". AD : Atrial Dimension; RV: Right Ventricle; LVMi: Left Ventricle Mass Index.

**Figure 2:**
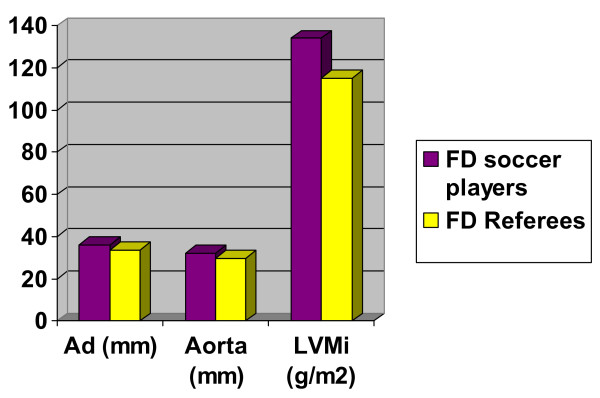
Histogram summarizing the echo-findings of the study in the two groups "FD referees vs FD soccer players". FD : First Division; AD : Atrial Dimension; RV: Right Ventricle; LVMi: Left Ventricle Mass Index

Each subject was examined in the morning after an overnight fast with mono-bidimensional echocardiographic and Doppler tests. The end-systolic and end-diastolic left ventricle diameters, interventricular septum and posterior wall thickness, left atrium and aorta with efflux tract measures were obtained with M-mode view starting from the long parasternal axis, and B- mode view according to the American Society of Echocardiography [5]. Fig. 1–2.

Left Ventricular Mass Index (LVMI) was calculated according to Devereux and Reichek [6]

using the formula:

LVM = [0.80 × 1.05 × (IVS + PW + Vsxd)^3 ^- Vsxd^3^)

Where:

- LVM = left ventricular mass in gr.

- IVS = measure of interventricular septum

- PW = measure of posterior wall

- Vsxd = left ventricular end-diastolic diameter.

The LVM value was normalized for body surface (g/m^2^). Diastolic function was evaluated with Doppler measurements on transmitral flow, and included E (early) and A (Atrial) peak mitral velocities, isovolumetric relaxation (IVR), and E/A ratio. All these variables were recorded from the four-chamber view[7].

### Statistical Analysis

Values were expressed as average ± standard deviation. Data were compared using an unpaired Student's t test. A *p *value of <0.05 was considered statistically significant

## Results

### Referees versus soccer players

No significant differences between referees and soccer players were found in: aortic root diameter, left ventricular diastolic dimension, left ventricular diastolic dimension, septum, posterior wall thickness. Left ventricular mass index (105.1 ± 19.3 g/m^2 ^vs 100.1 ± 45.2 g/m^2^), and right ventricle dimensions (22.2 ± 3.8 mm vs 25.9 ± 2.4 mm) were significantly greater in referees, left atrial dimensions (33.9 ± 7.4 mm vs 36.5 ± 4.5 mm) were greater in soccer players. Referees were significantly taller than soccer players. (Table 3, Fig. 1)

**Table 3:** Soccer players and referees

**General data**	**Soccers **N = 120	**Referees **N = 120
Age(years)	25.0 ± 13.0	32.0 ± 3.3
Height (cm)	172.5 ± 10.5*	184 ± 5.7 *
Weight(Kg)	65 ± 10.0	77.2 ± 6.6
HR(bpm)	72.2 ± 12.6	59.4 ± 13.5
SBP(mmHg)	119.5 ± 10.4	120.7 ± 13.2
DBP (mmHg)	76.0 ± 5.0	73.1 ± 10.2
**Echocardiographic data**		
Ad (mm)	36.5 ± 4.5*	33.9 ± 7.4*
IVS (mm)	9.8 ± 1.2	9.4 ± 0.6
PWT (mm)	9.6 ± 1.4	9.1 ± 0.6
Aorta (mm)	30.6 ± 4.4	29.6 ± 6.6
RV(mm)	22.2 ± 3.8*	25.9 ± 2.4 *
LVDD (mm)	50.7 ± 3.3	52.5 ± 3.5
LVDS (mm)	31.0 ± 3.0	32.5 ± 2.8
E peak (m/sec)	80.0 ± 15.0	80.9 ± 24.1
A peak (m/sec)	40.0 ± 15.0	48.4 ± 14.5
IVR (msec)	79.1 ± 14.8	68 ± 26.2
DT (msec)	192.5 ± 40.1	171.6 ± 54.5
LVM (g)	179.6 ± 81.4*	211.4 ± 37.1*
LVMi (g/m^2^)	100.5 ± 45.2*	105.4 ± 17.3*

HR heart rate, SBP systolic blood pressure, DBP diastolic blood pressure, Ad atrial dimension, IVS interventricular septum, PWT posterior wall thickness, RV right ventricle, LVDD left ventricular diastolic dimension, LVDS left ventricular systolic dimension, LVMi left ventricular mass index; *Significant difference according to Student's t test p < 0.05

### First-division referees versus first-division soccer players

The dimensions of the left atrium (33.7 ± 8.9 vs 36.2 ± 3.1), aortic root (29.7 ± 7.9 vs 32.1 ± 3) and LVMI (115.1 ± 16.7 vs 134.1 ± 19.9) were significantly greater in the soccer players than in referees. No differences in diastolic function were found between these two groups. Referees were significantly taller than soccer players. (Table 4, Fig. 2)

**Table 4:** First-division soccer players and first-division referees

**General data**	**FD Soccers **N = 35	**FD referees **N = 35
Age(years)	28.0 ± 6.7	34.3 ± 3.4
Height (cm)	180.8 ± 6.3*	184 ± 7.6 *
Weight(Kg)	78.8 ± 6.8	76.3 ± 8.2
HR(bpm)	59.3 ± 8.6	61.7 ± 16.9
SBP(mmHg)	120.5 ± 2.8	121.8 ± 13.0
DBP (mmHg)	75.9 ± 4.5	71.2 ± 13.7
**Echocardiographic data**		
Ad (mm)	36.2 ± 3.1*	33.7 ± 8.9*
IVS (mm)	10.6 ± 0.8	9.78 ± 0.6
PWT (mm)	10.2 ± 0.8	9.24 ± 0.6
Aorta (mm)	32.1 ± 3*	29.7 ± 7.9*
RV(mm)	24.7 ± 3.9	26.1 ± 2.5
LVDD (mm)	54.8 ± 3.2	54.2 ± 3.5
LVDS (mm)	34.1 ± 2.4	32.8 ± 2.7
E peak (m/sec)	74.8 ± 13.1	80.4 ± 30.2
A peak (m/sec)	41.7 ± 11.2	48.4 ± 11.7
IVR (msec)	75.8 ± 11.6	63.9 ± 22.6
DT (msec)	187.5 ± 35.9	157.4 ± 35.9
LVM (g)	268.5 ± 40.4	231 ± 37.1
LVMi (g/m^2^)	134.1 ± 19.9*	115.1 ± 16.7*

FD first division, HR heart rate, SBP systolic blood pressure, DBP diastolic blood pressure, Ad atrial dimension, IVS interventricular septum, PWT posterior wall thickness, RV right ventricle, LVDD left ventricular diastolic dimension, LVDS left ventricular systolic dimension, LVMi left ventricular mass index; *Significant difference according to Student's t test p < 0.05

## Discussion

Long-term athletic training is associated with morphological cardiac changes, including increased left ventricular cavity dimension, wall thickness, and calculated mass, which are commonly described as "athlete's heart".

These changes seem to be adaptations to the hemodynamic load produced by long-term, frequent, intensive exercise programs. Our investigation shows that referees' hearts present an increase in left ventricular mass and normal systolic and diastolic function, similar to that in soccer players. Thus a referee's heart can also be considered a physiological athlete's heart [8,9].

These morphological and functional modifications may be due to the fact that during the match and weekday training the work load is sufficiently intense to cause an increase in left ventricular mass, and morphological and functional modifications in the heart over the long term.

During a match in fact, a referee covers a distance of between 7000 and 10000 meters at an average heart rate of 150 to 160 bpm and an average oxygen uptake of approximately 80% of the maximum aerobic power. [10,11]

Elite soccer referees usually reach the peak of their careers at a considerably greater average age than competitive soccer players. Thus, referees perform best physically at a time when their cardiovascular performances start to decline.

So even if referees are older than soccer players, our echocardiographyc study shows how the referee's heart presents morphological adaptations like soccer, including similar parameters and an increase in left ventricular mass (both due to the training work load). For this reason the referee's cardiovascular adaptations allow the same soccer's players performance

The differences between first-division referees and elite soccer players may result from the different workloads undergone during both match and training. In fact, during an official match, soccer players cover a distance of between 8 and 12 km, with a speed of between 10 to19 Km/h, but their training load during the week is usually five days of training sessions plus the official match. The soccer players usually underwent training with a predominant increase in both volume endurance and pressure resistance exercise, while the referees mainly practiced endurance training [13].

This observation may explain the morphological differences between the players' and referees' left ventricular mass.

The present results are in partial disagreement with those of Castagna et al. [14,15], who studied the performances of referees of different age-groups and reported no differences in the 12-minute running test, whereas speed and acceleration tests were significantly better in the younger referees. However, the focus of the present investigation is on cardiac morphology at rest and systolic and diastolic function.

Finally, general performance in athletes is due not only to the contractile function of the heart, but also to complex interactions with the neurological and muscle-skeletal systems.

Our unpublished personal data on élite soccer players (ACF Fiorentina Football Team) have in fact showed that functional performances differed even among players whose cardiac mass was equal. The athletes followed the same training program.

## Conclusion and limitations of the study

This investigation has focused exclusively on cardiac morphology, and thus presents no functional correlation with maximum oxygen consumption and endurance, speed and strength performances.

Athletes between 35 and 40 years can maintain a good performance in both short- and long- distance running. However, even if these important parameters progressively decrease with age, with peak decrease occurring at about 50 years, performance is not dramatically impaired when the athlete is 40 years old. [16,17].

These results are supported in the literature by the study of Rotoyannis and Korhonem, who demonstrated that 35–40 year-old athletes are able to maintain good performance in marathon runs up to 50 Km, or sprints up to 100 m [18-21]

Finally, it would be interesting to conduct a longitudinal study on referees entering the first division. It is in fact important that a referee's physical performance should not decrease, in order also to guarantee the psychological aspects of refereeing, which are certainly improved with specific experience within the sporting context.

## Competing interests

The author(s) declare that they have no competing interests.

## Authors' contributions

The initial idea for the study was of GG. GG, AP, ML, LS and AM designed the study, and GG, LS, LT and AM performed all the measurements and statistical analyses. GG wrote the manuscript and all the authors contributed to, read, and approved the final version.

**Figure 3:**
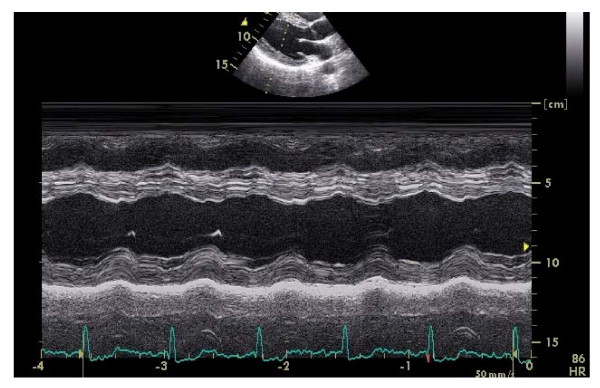
Soccer player 's 2D-targeted M-mode tracing.

**Figure 4:**
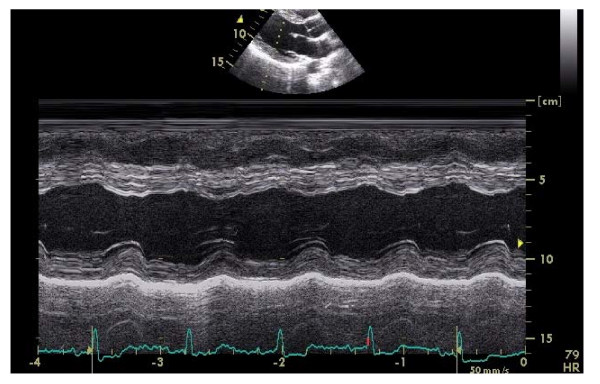
Referee's 2D-targeted M-mode tracing.
